# Depression and Its Correlates Among Brazilian Immigrants in Massachusetts, USA

**DOI:** 10.1007/s10903-017-0632-2

**Published:** 2017-07-31

**Authors:** Felippe Lazar-Neto, Andressa C. Sposato Louzada, Ricardo Faé de Moura, Fernando Morelli Calixto, Marcia C. Castro

**Affiliations:** 1 University of São Paulo Medical School, São Paulo, SP Brazil; 2 Faculty of Medical Sciences, Santa Casa of São Paulo, São Paulo, SP Brazil; 3 Department of Global Health and Population, Harvard T.H. Chan School of Public Health, 665 Huntington Avenue, Building I, Room 1113, Boston, MA 02115 USA

**Keywords:** Depression symptoms, CES-D, Brazilian immigrants

## Abstract

The goal of this paper is to assess the frequency of depression symptoms among Brazilian immigrants living in Massachusetts, the second largest Brazilian immigrant population in the United States, and to identify correlates of depression. A convenience sample of Brazilian immigrants aged 18 or older residing in Massachusetts was used. Data were collected from December 2013 to March 2014, in the Consulate General of Brazil in Boston and in three religious events, using a structure questionnaire and the Center for Epidemiological Studies Depression Scale (CES-D). Depression symptoms were observed in 35.3% of the respondents, with equal distribution by sex. Correlates of depression were low income, being single, poor English proficiency, and poor self-perception of health. These results suggest a need for community outreach, sensitization, and counseling, in Portuguese and adapted to the culture of Brazilian immigrants.

**Electronic supplementary material:**

The online version of this article (doi:10.1007/s10903-017-0632-2) contains supplementary material, which is available to authorized users.

## Introduction

Depression affects more than 120 million people worldwide [1] with a lifetime prevalence of about 10–15% [2]. In the 1990 and 2000 Global Burden of Disease (GBD) studies, depressive disorders were the fourth and third, respectively, leading cause of burden, and in 2000 it was the leading cause of disability [1]. In the 2010 GBD, the methodology was modified and the burden was measured separately for major depressive disorder (MDD) and dysthymia. MDD was the eleventh cause of burden, the second leading cause of disability, and a 37.5% increase in the years of life lived with disability was observed between 1990 and 2010, entirely accounted by population growth and ageing [3]. The burden of depression is multi-fold; it affects individual overall mortality, increases the risk of suicide, and of mortality from cardio and cerebrovascular disease; it may lead to individual impairment, problems with social interaction, and family disruption; it affects fetal, neonatal, and child development, in the case of maternal depression; and it results in workdays lost, impacting individual and national economics [1, 4, 5]. However, the burden of depressive disorders is not homogeneous by country/region and demographics [6].

Risk factors commonly associated with both prevalence and incidence of depression include chronic diseases, poor self-perceived health, functional disability, personality traits, inadequate coping strategies, previous psychopathology, limited and/or poor quality social networks, stressful life events, being unmarried, being a female, and socioeconomic status (low income and low education) [7, 8]. Larger prevalence of depression among women can be partially explained by a higher sensitiveness for adverse life events, social roles, and cultural norms [9]. At younger ages, correlates include illicit substance, alcohol abuse, quality of sleep [10], and body dissatisfaction [11]. Specifically related to immigrants and refugees in the United States (US), possible correlates of depression include cultural conflict, language proficiency, social isolation and poor social integration, low neighborhood safety, unauthorized immigration status, employer exploitation, exposure to discrimination, disrupted marital status, unemployment, perceived low social standing, time of residence in the US, lack of health insurance, pre-migration factors, and individual health practices [12–16].

According to the 2010–2014 American Community Survey (ACS) [11], there were 41 million foreign-born people in the US (almost 13% of the total population), and 52% of those were from Hispanic or Latino origin. Brazilian immigrants, in particular, amounted to 334,511 people, and Massachusetts (MA) was the state with the second largest Brazilian immigrant population (60,790), after Florida (66,977). This number is thought to be underestimated due to concerns about survey confidentiality among those with unauthorized immigration status and to confusion in declaration of Brazilian ethnicity under US-defined categories [17]. Data from a 2007 survey suggests that 71% of adult Brazilian immigrants living in the Boston–Cambridge–Quincy metropolitan area and 16% of their children were undocumented [17].

Ethnic minorities are likely to experience disparities in access and quality of mental health services [15, 18]. Several studies focused on Latinos and depression in the US, but often they did not include Brazilians [15, 16, 19, 20]. Indeed, analyzing Brazilian immigrants separate from Latinos is an important distinction due to different cultural background (Brazil was a Portuguese colony, heavily influenced by African culture due to the slave trade), and language (Portuguese vs. Spanish) [15, 21]. Available evidence suggests that Brazilian immigrants may be exposed to varied job hazards, including psychosocial, resulting in sleeping problems and periods of depression [22]. In addition, English language fluency and cultural differences awareness were found to be important predictors of good quality mental health care delivery to the Brazilian immigrant population [23]. Marcelli et al. [17] suggested that Brazilian immigrants in the greater Boston area were healthier than US-born residents, except with regards to mental health, partially related to the pressure of lack of documentation [17]. Yet, a comprehensive study that attempts to shed light on the correlates of depression symptoms among Brazilian immigrants in the US is not available.

The goals of this paper are three-fold: (i) to assess how frequent is the presence of depression symptoms among sampled Brazilian immigrants living in MA, (ii) to identify correlates of depression in this population, and (iii) to use the results to propose recommendations to mitigate the problem. Our results contribute to extend knowledge of potential challenges regarding the mental health of Brazilian immigrants.

## Methods

### Measures

In order to gather detailed information from the population of Brazilian immigrants in MA a convenience sample survey was conducted from December 2013 to March 2014. To the best of our knowledge, this was the first survey that targeted only Brazilians in MA, with a focus on chronic diseases and depression. To facilitate comparisons of risk factors with the Brazilian population living in Brazil, the survey instrument was based on a nationwide telephone survey launched in 2006 and annually conducted in Brazil titled Surveillance of Risk and Protective Factors of Chronic Diseases (*Vigilância de Fatores de Risco e Proteção para Doenças Crônicas por Inquérito Telefônico* - VIGITEL) [24]. To assess specific variables related to immigrants we added questions on time living in the US and in MA, personal income, changes in nutritional intake after moving to the USA, and English language proficiency. In addition, to measure the occurrence of depression symptoms we added the CES-D scale composed of 20 questions that often cover four major structure factors: positive affects; depressive affects; somatic and retarded activity; and interpersonal. The CES-D scale has been translated, validated and applied in different countries [25–27] including Brazil [28]. Therefore, we used the validated Portuguese version that considers as depressed all individuals with a score equal to or greater than 15 (100% sensibility and 75% specificity) [29].

### Data Collection

A small number of the interviews (15%) were conducted during events in three specific churches in Somerville, Cambridge and Framingham, following religious masses regularly held for the Brazilian immigrant community. The majority (85%) of interviews, however, were conducted at the Consulate General of Brazil in Boston, among Brazilians who searched for varied consular services. The concentration of interviews at the Consulate sought to obtain better representativeness of the immigrant population, since people from diverse demographic characteristics, socioeconomic background, and place of residence in MA regularly seek information and varied services at the Consulate (e.g., issuing of documents; renewal of personal identification card and voting card; transfer of voting rights; registration of births, deaths, and weddings; voting for elections; overall counseling; etc.).

### Participants

Eligibility criteria were: (i) birth in Brazil; (ii) aged 18 years or over; and (iii) current residence in MA. All interviews were conducted in Portuguese by Brazilian medical students. Upon consent, the survey questionnaire was handed over to the participant, who answered the questions without interference of the interviewer. A total of 521 interviews were conducted utilizing the VIGITEL-based structured questionnaire and the CES-D scale. Of those, 401 answered all 20 questions of the CES-D scale, and constitute the sample for this study. The remaining 120 provided partial or no answer to the CES-D scale module, despite having answered other questions of the questionnaire. This lack of response may indicate refusal to answer the CES-D module; however, since the questionnaire was self-administered, interviewers did not approach subjects to find out reasons of non-response.

### Analysis

Reliability of the CES-D scale was assessed through an analysis using the Chronbach’s alpha. Following CES-D protocol [30], individual scores were calculated based on the responses to the 20 questions included in the scale. A binary variable was created to indicate if individuals presented depression symptoms (CES-D scores ≥15), and descriptive statistics were computed to characterize the Brazilian immigrants with and without depression symptoms living in MA.

In order to identify correlates of depression among Brazilian immigrants the following covariates were included: age (18–34; 35–49; ≥50 years); place of interview (churches = 0; consulate = 1); time of residence in the US (<12 years, ≥12 years); time of residence in the city (<9 years; ≥9 years); English language proficiency (fluent, good, regular/poor); monthly income in US dollars (>$3500; $1500–$3500; $600–$1500 monthly); health insurance coverage (a binary variable, 1 = yes); residence in a place with nearby public space to exercise (a binary variable, 1 = yes); sex (female = 1); self-perception of health (very good, good, regular/bad); marital status (single, married/civil union, divorced/widowed); education (primary school; high school; university; graduate school); smoking habits (a binary variable, 1 = no); work in the past 3 months (a binary variable, 1 = no); alcohol consumption (a binary variable, 1 = no); presence of a chronic disease such as diabetes or/and hypertension (a binary variable, 1 = yes); reproductive years for females (0 = >49 years, 1 = 18–49 years); have exercised in the past 3 months (a binary variable, 1 = yes); and weight self-perception (under the ideal, ideal, over the ideal). In addition, based on self-reported information on height and weight, the body mass index (BMI) was calculated, and a binary variable indicating overweight/obesity (values of BMI ≥25 kg/m^2^) was created [31]. Differences in the categorical variables between groups with and without depression symptoms were analyzed using Chi square tests.

Univariate logistic regressions were run for each covariate against the depression indicator variable. Interactions for sex were also run considering the evidence in the literature of differences in depression between males and females. All covariates that were significant in the univariate models were included in a multivariate logistic regression. Model goodness-of-fit was assessed through the calculation of three diagnostics measures: (i) the area under the ROC (Receiver Operating Characteristic) curve; (ii) the detection of extreme observations (Pearson residuals, standardized residuals, deviance, and Pregibon leverage); and (iii) the assessment of heteroscedasticity in the residuals (robust standard errors). All statistical analyses were done in STATA v.13 (Stata Corp.; College Station, TX, USA).

### Human Subject Protection Approval

The Harvard T. H. Chan School of Public Health Institutional Review Board approved the study by Protocol 13-2450.

## Results

The coefficient of internal consistency for the CES-D for total scores was 0.84. Question number four, “When comparing myself with other people I felt I was as good as most of them” had a low item-total correlation coefficient (r < 0.15). With the exclusion of this question from the total composite, the coefficient increased to 0.88. This result is consistent with the Brazilian CES-D scale validation study [28].

The characteristics of individuals included in the study are presented in Table 1. The mean age was 38.7 years, and 56% of study participants were women. The presence of depression symptoms (CES-D score ≥15) in the overall sample was 35.3%, with roughly equal distribution for males (35.3%) and females (35.0%). The average time that participants were living in the US was 12.3 years, and in the current city it was 9.15 years. Significant differences between individuals with and without depression symptoms were observed by marital status, income, time lived in the US, English proficiency, self-perception of health, and health insurance status.

**Table 1:** Characteristics of study participants stratified by the presence of depression symptoms based on CES-D score

Variables	Total subjects (% in the sample)		CES-D Score				χ^2a^
			CES-D <15 (% in the line)		CES-D ≥15 (% in the line)		
Marital status							15.330***
Single	92	(23.1%)	45	(48.9%)	47	(51.1%)	
Married/civil union	259	(64.9%)	185	(71.4%)	74	(28.6%)	
Divorced/widowed	48	(12.0%)	30	(62.5%)	18	(37.5%)	
Monthly income (US$)							19.674***
>$3500	97	(26.2%)	81	(83.5%)	16	(16.5%)	
$1500–$3500	165	(44.6%)	102	(61.8%)	63	(38.2%)	
<$1500	108	(29.2%)	60	(55.6%)	48	(44.4%)	
English language proficiency							14.251***
Fluent	148	(37.0%)	109	(73.7%)	39	(26.4%)	
Good	108	(27.0%)	75	(69.4%)	33	(30.6%)	
Regular, bad or do not speak	144	(36.0%)	77	(53.5%)	67	(46.5%)	
Self-perception of health							9.256**
Very good	105	(26.5%)	79	(75.2%)	26	(24.8%)	
Good	197	(49.6%)	128	(70.0%)	69	(35.0%)	
Regular or worst	95	(23.9%)	52	(54.7%)	43	(45.3%)	
Age							1.455
18–34 years	151	(38.7%)	97	(64.2%)	54	(35.8%)	
35–49 years	173	(44.4%)	109	(63.0%)	64	(37.0%)	
≥50 years	66	(16.9%)	47	(71.2%)	19	(28.8%)	
Has health insurance							4.785*
Yes	328	(83.7%)	221	(67.4%)	107	(32.6%)	
No	64	(16.3%)	34	(53.1%)	30	(46.9%)	
Access to public space							1.290
Yes	340	(86.5%)	226	(66.5%)	114	(33.5%)	
No	53	(13.5%)	31	(58.5%)	22	(41.5%)	
Sex							0.004
Man	173	(43.4%)	112	(64.7%)	61	(35.3%)	
Woman	226	(56.6%)	147	(65.0%)	79	(35.0%)	
BMI							0.191
Normal	193	(54.2%)	126	(65.3%)	67	(34.7%)	
Overweight/obese	163	(45.8%)	110	(67.5%)	53	(32.5%)	
Weight self-perception							3.282
Ideal	209	(25.6%)	140	(67.0%)	69	(33.0%)	
Under	12	(3.0%)	5	(41.7%)	7	(58.3%)	
Over	176	(44.3%)	113	(64.2%)	63	(35.8%)	
Education							4.731
Primary school	63	(15.8%)	35	(55.6%)	28	(44.4%)	
High school	147	(36.9%)	93	(63.3%)	54	(36.7%)	
University	137	(34.4%)	94	(68.6%)	43	(31.4%)	
Graduated	51	(12.8%)	37	(72.6%)	14	(27.5%)	
Smoking							0.168
Yes	29	(7.4%)	20	(69.0%)	9	(31.0%)	
No	365	(92.6%)	238	(65.2%)	127	(34.8%)	
Worked in the past 3 months							0.004
Yes	364	(92.2%)	237	(65.1%)	127	(34.9%)	
No	31	(7.9%)	20	(64.5%)	11	(35.5%)	
Alcohol consumption							1.190
Yes	184	(46.8%)	125	(67.9%)	59	(32.1%)	
No	209	(53.2%)	131	(62.7%)	78	(37.3%)	
Has a chronic disease							1.133
Yes	59	(14.7%)	42	(71.2%)	17	(36.0%)	
No	342	(85.3%)	219	(64.0%)	123	(28.8%)	
Exercised in the past 3 months							0.0316
Yes	259	(65.1%)	170	(65.6%)	89	(34.4%)	
No	139	(34.9%)	90	(64.8%)	49	(35.3%)	
Time in US							10.525***
<12 years	185	(46.5%)	105	(56.8%)	80	(43.2%)	
≥12 years	213	(53.5%)	154	(72.3%)	59	(27.7%)	
Time in city							2.495
<9 years	189	(48.2%)	115	(60.9%)	74	(39.2%)	
≥9 years	203	(51.8%)	139	(68.5%)	64	(31.5%)	
Women by age group							0.7932
>49 years	35	(16.0%)	25	(71.5%)	10	(28.6%)	
18–49 years	184	(84.0%)	117	(63.6%)	67	(36.4%)	

^a^Chi-square test, *p < 0.05; **p ≤ 0.01; ***p ≤ 0.001

In order to assess if subjects interviewed at the consulate and churches were significantly different, we compared their characteristics (Supplemental Table S1). No significant differences were found except for age, time living in the US, and health insurance: those attending churches were, on average, older, had lived in the US for a longer period of time, and had better health insurance coverage. In addition, to assess if significant bias in migrants’ characteristics existed in our sample, we compared descriptive statistics from our data (Table 1) with the 2014 ACS using “Massachusetts” and “Brazilians” as a filter (Supplemental Table S2). Considering that for some variables the age groups were different in the two surveys (e.g., for marital status, our sample includes only subjects 18 or older, while the ACS considered 15 or older), our sample had several characteristics similar to the ACS. In particular, we found similar distributions in sex, age, and time living in the US, but notable differences regarding health insurance, education, and employment status were observed.

**Fig. 1:**
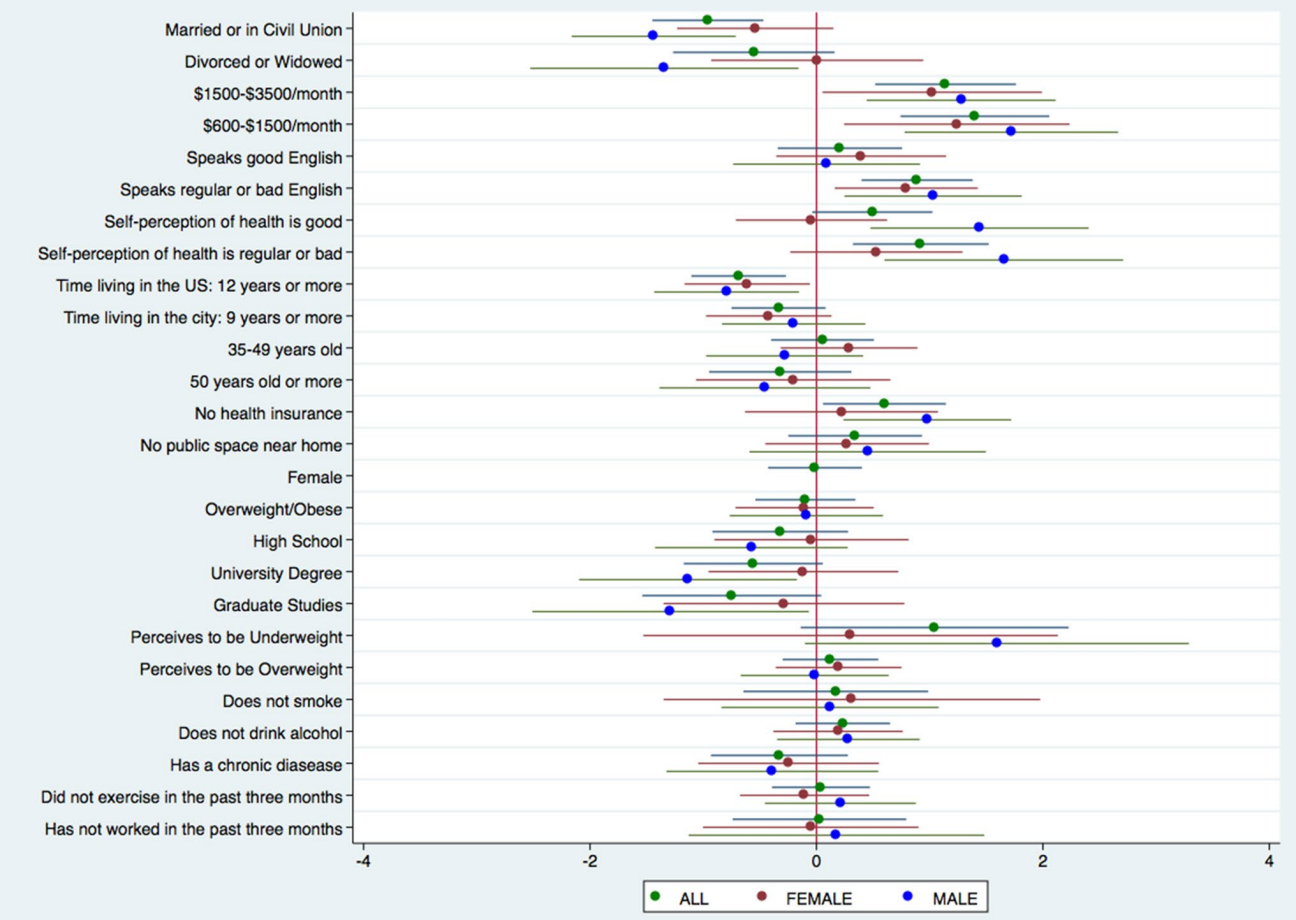
Univariate logistic regression models on the presence of depression symptoms in the entire sample, and stratified by sex. *Bars* across each coefficient point estimate represent the 95% confidence interval.

Figure 1 shows the results of univariate logistic regression between depression symptoms and each selected covariate. Based on previous findings of sex differences in depression, univariate models stratified by sex were also run. Significant results were observed for marital status (particularly for males), monthly income, English language proficiency, self-perception of health (particularly for males), time living in the US, and health insurance plan (particularly for males). In addition, an interaction term between each variable and sex revealed significant effect modification for marital status and self-perception of health (results not shown). Place of interview was not associated with depression symptoms (p = 0.772). Considering only women, being in the reproductive age period (18–49 years) was associated with higher odds of having depression (odds ratio [OR] 1.43; 95% confidence intervals [95% CI] 0.64–3.16); however, this result was not statistically significant.

Table 2 shows the results of multivariate logistic regressions. Model 1 included all variables that were significant in the univariate models, and Model 2 added sex-interaction terms. Although age was not significant in the univariate model, we included it in the model to control for any potential time effects not captured by the duration of residence in the US.

**Table 2:** Multivariate logistic regression models on the presence of depression symptoms

Variables	Model (1)		Model (2)	
	OR	95% CI	OR	95% CI
Marital status				
Single	1.00		1.00	
Married/civil union	0.32	(0.17–0.60)	0.12	(0.04–0.34)
Divorced/widowed	0.49	(0.20–1.17)	0.12	(0.02–0.58)
Married/civil union women			4.96	(1.36–18.14)
Divorced/widowed women			7.99	(1.20–53.24)
Monthly income (US$)				
>$3500	1.00		1.00	
$1500–$3500	3.07	(1.49–6.31)	3.03	(1.43–6.43)
<$1500	3.05	(1.40–6.67)	3.07	(1.37–6.90)
English language proficiency				
Fluent	1.00		1.00	
Good	1.90	(0.95–3.80)	1.99	(0.98–4.05)
Regular, bad or do not speak	2.37	(1.17–4.80)	2.35	(1.13–4.88)
Self-perception of health				
Very good	1.00		1.00	
Good	1.33	(0.72–2.49)	5.87	(1.67–20.67)
Regular or worst	2.19	(1.08–4.47)	10.67	(2.57–44.22)
Good women			0.12	(0.03–0.51)
Regular or bad women			0.12	(0.02–0.63)
Age				
18–34 years	1.00		1.00	
35–49 years	1.54	(0.85–2.80)	1.54	(0.83–2.84)
≥50 years	0.91	(0.38–2.14)	0.97	(0.40–2.35)
Has health insurance	1.27	(0.62–2.57)	1.41	(0.65–3.03)
Women	0.98	(0.57–1.67)	1.61	(0.38–6.78)
Has lived ≥12 years in the US	0.60	(0.33–1.08)	0.62	(0.34–1.15)
Education				
Primary school	1.00		1.00	
High school	0.75	(0.36–1.55)	0.90	(0.42–1.94)
University	0.92	(0.41–2.04)	1.04	(0.45–2.42)
Graduate school	0.72	(0.24–2.08)	0.94	(0.31–2.88)
ROC	0.7462		0.7780	

*OR* odds ratio, *CI* confidence intervals

Compared to individuals who were single, being married or in a civil union reduced the odds of having depression symptoms by 68% (OR 0.32; 95% CI 0.17–0.60). However, after accounting for an interaction between marital status and sex (Model 2), significant effects were observed only for males (OR 0.12; 95% CI 0.04–0.34). Income was a strong predictor of depression symptoms, since immigrants who made less than US$3500 per month had about three times the risk of depression compared with those who made more than US$3500 per month. Regarding proficiency in English, an important factor for social acculturation, compared with individuals who could speak fluent English, those who had difficulties in communicating were 2.37 (OR 2.37; 95% CI 1.17–4.80) times more likely to present depression symptoms. Individual perception of health was also important, and the odds of having depression symptoms for individuals who reported regular or bad health conditions were 119% (OR 2.19; 95% CI 1.08–4.47) higher than those who viewed their health status as very good. Similarly to marital status, after considering an interaction between perception of health and sex, effects were only significant for men (OR 10.66; 95% CI 2.57–44.22).

Model diagnostics indicated good predictive power, as represented by a ROC value of 0.75 and 0.78 for Models 1 and 2, respectively (Table 2). In addition, a robust estimation did not change significantly the standard errors, and therefore heteroscedasticity was not likely to occur in the models. Sensitivity of the model was assessed by removing eight observations with extreme values (large residuals) in Model 1, and nine observations in Model 2. The new fitted models indicated no significant changes in the magnitude of estimated coefficients.

## Discussion

Our results showed that a little over a third of the Brazilian immigrants included in the study presented depression symptoms, which prevalence was roughly the same for males and females. Important correlates of depression included low income, being single, poor English proficiency, and poor self-perception of health. Marital status and self-perception of health were significant only for males.

The observed occurrence of depression symptoms in our sample is almost ten times higher than the prevalence of depression reported in nationally representative household surveys conducted in Brazil in 2003 and 2008 [32]. However, those surveys were based on self-reported data, not on standardized instruments such as the CES-D. Therefore, the results from Brazil could be underreported. Although there is evidence that female prevalence of depression tends to be higher in the general population, especially during the reproductive years [33], our results did not show significant differences by sex. Indeed, the association between sex and depression has shown conflicting results in studies that focused on immigrants [12, 34, 35].

Lower income can affect the odds of having depression through different mechanisms, including (i) it imposes stress on the individual’s ability to pay bills (e.g., rent, utilities, food, transportation); (ii) it might restrict leisure activities; and (iii) it might limit the chances that an individual will have health insurance, altering health care seeking behavior, and decreasing the probability of proper treatment [1]. Our univariate analysis showed that lack of health insurance was associated with depression symptoms, but results were not significant in the multivariate analysis. It is likely that the income variable captured the potential effects of health coverage.

A more complex difference emerges regarding marital status and depression symptoms. Previous studies have shown that marriage was a protective factor for the mental health of Latino immigrants [12, 36], however sex differences were not assessed in those studies. Our study showed that marriage was protective for men but not for women. Forty years ago, sociologist Jessie Bernard proposed that marriage would be beneficial only for men, mainly due to social roles played by both sexes at that time [37]. A longitudinal study that assessed sex-specific differences in marriage found that it was associated with lower levels of depression among men, but not among women [19]. In addition, a recent meta-analysis showed that divorced, widowed, and never married people had higher odds of developing depression later in life when compared to married counterparts, independently of sex [38]. More research is needed to identify the extent to which the effect of marital status on depression for immigrants of different ethnicities varies between males and females.

Lack of proficiency in English brings about major barriers to social interaction, local adaptation, and types of jobs Brazilian immigrants can have. In addition, health care can be compromised in those cases due to poor communication between health providers and patients, unless a Brazilian Portuguese interpreter mediates the patient-provider interaction. This is of particular concern for the treatment of depression, since specific cultural expressions to describe feelings and emotions in Portuguese may not have a straightforward English translation [21].

Poor health perception has been associated with depression symptoms in several contexts [20, 21, 31, 32]. The findings observed in this study could be partially explained by differences in help-seeking behavior, since females are more likely to report physical and mental health symptoms and to seek medical help [9], thus making the self-perception of health less specific for mental health problems among women.

This study has some limitations. The interviews were conducted from November to April, when average temperatures are low, which could be challenging for Brazilians, who are accustomed to warmer climates. Thus, it is possible that our results overestimate the frequency of depression symptoms, as seasons can affect mood and increase depression symptoms in susceptible people [39]. We do not expect that the correlates of depression would significantly vary by season. Because we did not inquire participants about their legal status in the US, we are not able to assess whether or not Brazilian undocumented immigrants have significantly higher prevalence of depression symptoms than documented ones. Reasons for seeking out service at the Consulate were not inquired, and thus we were not able to distinguish individuals who were under distress and, therefore, more likely to present depressive symptoms. Lastly, although English proficiency could be a proxy to acculturation or bi-cultural competence, this was not directly measured.

This study highlights that the presence of depression symptoms among our sample of Brazilian immigrants in Massachusetts is high. Although the size of the Brazilian immigrant community in MA is substantial, this population remains understudied, often not included in Latino immigration studies. Our results suggest a need for community outreach, sensitization, and counseling, adapted to the culture and the language of Brazilian immigrants. A few resources are provided by the state, such as a Multicultural Populations Resource Directory, made available by the Office of Multicultural Affairs, of the Department of Mental Health. This directory provides information on services available through many organizations and associations. There are only a handful of groups that target Portuguese speakers, and only one of those provides mental health services. A specific model that could be considered in this context is the System Integrative Model for Community Therapy [38]. This model, created by Brazilian anthropologist Adalberto Barreto, is a shift from the common provision of services heavily focused on medications, to a model that values community participation, empowerment, and peer support [38]. Mitigating the frequency of depression symptoms would be beneficial to Brazilian immigrants, their families, and communities where they work and study, as well as the American society.

### New Contribution to the Literature

The size of the Brazilian immigrant community in MA is substantial; yet, this remains an understudied population, often not included in Latino immigration studies. Here we present the frequency of depression symptoms among Brazilian immigrants in MA, and discuss their correlates. Mitigating the occurrence of depression symptoms would be beneficial to individuals, to their families, to communities where they work/study, and to the American society.

## Electronic supplementary material

Below is the link to the electronic supplementary material.

*Title of data* Dataset of Brazilian Immigrants in Massachusetts included in the study sample. *Description of data* Dataset with all variables used in the study. (XLSX 79 KB)

The table shows proportions and Chi square tests for variables considered in the analysis, stratified by place of interview. (DOCX 20 KB)

This table shows the proportions and respectively confidence intervals of the following variables for both samples: marital status, English proficiency, age groups, sex, education attainment, unemployment rate, time of residence in the US, health insurance and income. (DOCX 17 KB)

## Abbreviations

ACS: American Community Survey
CES-D: Center for Epidemiological Studies Depression Scale
OR: Odds ratio
CI: Confidence interval
GBD: Global burden of disease
MDD: Major depressive disorder
MA: Massachusetts
VIGITEL: Vigilância de Fatores de Risco e Proteção para Doenças Crônicas por Inquérito Telefônico
BMI: Body mass index
ROC: Receiver operating characteristic
