# Supplementary material for: Depression and Its Correlates Among Brazilian Immigrants in Massachusetts, USA
Source: J Immigr Minor Health. 2017 Jul 31;20(4):832–40. doi: 10.1007/s10903-017-0632-2 (PMC6061077; doi:10.1007/s10903-017-0632-2)
Supplement: Supplementary file 2 — The table shows proportions and Chi square tests for variables considered in the analysis, stratified by place of interview. (DOCX 20 KB) [file 10903_2017_632_MOESM2_ESM.docx]

**Table S1 – Descriptive statistics of subjects interviewed at the Consulate General of Brazil in Boston and at Churches**

| **Characteristics** | **Churches** | | **Consulate** | | **χ2 †** |
| --- | --- | --- | --- | --- | --- |
|  | **#** | **Percentage** | **#** | **Percentage** |  |
| *Marital Status* |  |  |  |  | 3.007 |
| Single | 23 | 29% | 95 | 22% |  |
| Married/Civil Union | 44 | 56% | 290 | 66% |  |
| Divorced/Widowed | 11 | 14% | 52 | 12% |  |
| *Income* |  |  |  |  | 1.438 |
| >$3500 | 17 | 23% | 103 | 26% |  |
| $1500-$3500 | 34 | 46% | 174 | 43% |  |
| <$1500 | 23 | 31% | 125 | 31% |  |
| *English* |  |  |  |  | 6.546 |
| Fluent | 27 | 34% | 138 | 32% |  |
| Good | 28 | 35% | 108 | 25% |  |
| Regular, Bad or Do not Speak | 24 | 30% | 191 | 44% |  |
| *Age* |  |  |  |  | 24.911*** |
| 18-34 years | 11 | 14% | 181 | 42% |  |
| 35-49 years | 42 | 54% | 176 | 41% |  |
| >= 50 years | 25 | 32% | 70 | 16% |  |
| *Sex* |  |  |  |  | 0.914 |
| Men | 31 | 40% | 203 | 46% |  |
| Women | 46 | 60% | 237 | 54% |  |
| *Education* |  |  |  |  | 2.149 |
| Primary School | 13 | 16% | 98 | 23% |  |
| High School | 32 | 41% | 158 | 36% |  |
| University | 23 | 29% | 131 | 30% |  |
| Graduated | 11 | 14% | 46 | 11% |  |
| *Time in US* |  |  |  |  | 6.230* |
| <12 years | 26 | 33% | 213 | 49% |  |
| >= 12 years | 52 | 67% | 225 | 51% |  |
| *CES-D score* |  |  |  |  | 0.084 |
| <15 | 40 | 63% | 221 | 65% |  |
| >=15 | 23 | 37% | 117 | 35% |  |
| *CES-D score if men* |  |  |  |  | 0.785 |
| <15 | 13 | 57% | 99 | 66% |  |
| >=15 | 10 | 43% | 51 | 34% |  |
| *CES-D score if women* |  |  |  |  | 0.0112 |
| <15 | 25 | 66% | 122 | 65% |  |
| >=15 | 13 | 34% | 66 | 35% |  |
| *Health Self-Perception* |  |  |  |  | 1.562 |
| Very Good | 26 | 34% | 113 | 27% |  |
| Good | 34 | 44% | 203 | 48% |  |
| Regular or Worst | 17 | 22% | 105 | 25% |  |
| *Insurance* |  |  |  |  | 4.2333* |
| Yes | 69 | 91% | 329 | 81% |  |
| No | 7 | 9% | 77 | 19% |  |
| *Working* |  |  |  |  | 2.723 |
| Yes | 75 | 97% | 389 | 92% |  |
| No | 2 | 3% | 33 | 8% |  |
| *Time in City* |  |  |  |  | 0.365 |
| <9 years | 38 | 50% | 197 | 46% |  |
| >= 9 years | 38 | 50% | 229 | 54% |  |
| † = Chi-square test; *p < 0.05; ** p ≤ 0.01; *** p ≤ 0.001. | | | | | |
