# Supplementary material for: Depression and Its Correlates Among Brazilian Immigrants in Massachusetts, USA
Source: J Immigr Minor Health. 2017 Jul 31;20(4):832–40. doi: 10.1007/s10903-017-0632-2 (PMC6061077; doi:10.1007/s10903-017-0632-2)
Supplement: Supplementary file 3 — This table shows the proportions and respectively confidence intervals of the following variables for both samples: marital status, English proficiency, age groups, sex, education attainment, unemployment rate, time of residence in the US, health insurance and income. (DOCX 17 KB) [file 10903_2017_632_MOESM3_ESM.docx]

**Table S2 – Descriptive Statistics of the Sample used in this study and of the 2014 American Community Survey**

| **Characteristics of the survey** | **Percentage (Confidence Interval)** | |
| --- | --- | --- |
|  | **American Community Survey 2014** | **Survey used in this study** |
| *Marital Status** |  |  |
| Single | 32.4% (27.9-36.9) | 22.91% (19.47-26.75) |
| Married/Civil Union | 53.1% (47.3-58.9) | 64.85% (60.61-68.87) |
| Divorced | 8.0% (7.6-10.4) | 12.23% (9.66-15.36) |
| Widowed | 1.2% (0.2-2.2) |  |
| *English*** |  |  |
| Less than good | 43% (36.8-49.2) | 41.67% (37.47-45.98) |
| *Age*§ |  |  |
| Under 5 years | 12.6% (9.6-15.6) |  |
| 5-17 years | 19.6% (16.5-22.7) |  |
| 18-24 years | 5.4% (3.6-7.2) | 8.11% (6.02-10.85) |
| 25-34 years | 22.2% (18.2-26.2) | 29.9% (26.05-34.05) |
| 35-44 years | 20.4% (17.3-23.5) | 31.49% (27.56-35.68) |
| 45-54 years | 10.6% (8.00-13.2) | 20% (16.72-23.73) |
| 55-64 years | 8% (5.2-10.8) | 8.11% (6.02-10-85) |
| 65-74 years | 1.0% (0.4-1.6) | 2.18% (1.20-3.89) |
| >75 years | 0.2% (0.00-0.4) | 0.19% (0.02-1.40) |
| *Sex* |  |  |
| Man | 45.6% (41.6-49.3) | 45.26% (41.00-49.59) |
| Woman | 54.4% (50.7-58.1) | 54.74% (50.40-58.99) |
| *Education**** |  |  |
| Primary School | 15.6% (11.5-19.7) | 21.68% (18.31-25.47) |
| High School | 50.3% (44.6-56) | 37.11% (33.01-41.39) |
| Some college | 21.3% (16.3-26.3) | 30.08% (26.24-34.20) |
| Bachelor degree | 10.0% (6.7-13.3) |  |
| Graduated | 2.8% (1.6-4.0) | 11.13% (8.67-14.17) |
| *Working* |  |  |
| Employed | 78.1% (74.3-81.9) | 92.99% (90.37-94.92) |
| Unemployed | 5.4% (3.4-7.4) | 7.01% (5.07-9.62) |
| *Time in US* |  |  |
| Entered 2010 or later | 11.7% (6.8-16.6) | 11.05% (8.61-14.06) |
| Entered 2000 - 2010 | 56.0% (49.7-62.3) | 58.53% (54.20-62.71) |
| Entered before 2010 | 32.3% (27.1-37.5) | 30.43% (26.59-34.54) |
| *Insurance* |  |  |
| No Insurance | 9.8% (6.9-12.7) | 17.43% (14.28-21.09) |
| *Income (monthly)* |  |  |
| $600-$1000 | $1.489/monthly**** | 19.54% (16.20-23.36) |
| $1000-$1500 |  | 11.55% (8.96-14.76) |
| $1500-$2500 |  | 24.79% (21.10-28.88) |
| $2500-$3500 |  | 18.91% (15.62-22.69) |
| >$3500 |  | 25.21% (21.49-29.32) |
| *ACS included 15 years and older | | |
| ** ACS included 5 years and old and the question was less than "Very Well" | | |
| *** ACS included people 25 years and older | | |
| **** Per capita | | |
| § Our sample did not include people under 18 years old | | |
